# Retraction: Overexpression of lncRNA ANRIL up-regulates VEGF expression and promotes angiogenesis of diabetes mellitus combined with cerebral infarction by activating NF-κB signaling pathway in a rat model

**DOI:** 10.18632/oncotarget.28572

**Published:** 2025-01-20

**Authors:** Bo Zhang, Dan Wang, Tie-Feng Ji, Lei Shi, Jin-Lu Yu

**Affiliations:** ^1^Department of Neurosurgery, The First Hospital of Jilin University, Changchun, P.R. China; ^2^Department of Ophthalmology, The First Hospital of Jilin University, Changchun, P.R. China; ^3^Department of Radiology, The First Hospital of Jilin University, Changchun, P.R. China; ^*^These authors have contributed equally to the manuscript

**This article has been retracted:** Oncotarget has completed its investigation of this paper. After corresponding author Jin-lu Yu contacted the journal requesting a retraction, Oncotarget contacted the President of The First Hospital, Jilin University, to initiate an investigation, but no reply was received. Oncotarget has since discovered several image overlaps with unrelated papers from different institutions. Specifically, Figure 5, panels B and C, contain IHC images that overlap with Figure 3A from an unrelated paper published slightly later [1]. Figure 6, representing results of immunostaining, contains overlaps in panels A and C, and panel A overlaps with Figure 6A in a paper accepted for publication shortly thereafter [2]. In addition, Figure 7, illustrating data of microvessel density measurement, contains image overlaps within panels B, D, and E, and internal overlaps in panel C. Moreover, many IHC images and images of the specimens of the cerebral infarction were reused in later published articles in other journals. In light of these facts, the Editorial decision has been made to retract this paper. All authors agreed with the decision.

Original article: Oncotarget. 2017; 8:17347–17359. 17347-17359. https://doi.org/10.18632/oncotarget.14468
